# ^68^Ga-NY104 PET/CT in the Differential Diagnosis of FDG-Negative Renal Masses: A Two-Case Illustration of Clear Cell Carcinoma Versus Renal Hemangioma

**DOI:** 10.3390/diagnostics15233049

**Published:** 2025-11-29

**Authors:** Xinchun Yan, Yichen Xie, Guoyang Zheng, Jingci Chen, Wenjia Zhu, Li Huo

**Affiliations:** 1 Department of Nuclear Medicine, Peking Union Medical College Hospital, Peking Union Medical College, Chinese Academy of Medical Sciences, Beijing 100730, China; daleyenxc@outlook.com (X.Y.); xycicey@outlook.com (Y.X.); 2 Department of Urology, Peking Union Medical College Hospital, Peking Union Medical College, Chinese Academy of Medical Sciences, Beijing 100730, China; guoyang198966@163.com; 3 Department of Pathology, Peking Union Medical College Hospital, Peking Union Medical College, Chinese Academy of Medical Sciences, Beijing 100730, China; chenjingci@pumch.cn

**Keywords:** clear cell renal cell carcinoma, carbonic anhydrase IX, positron emission tomography, computed tomography

## Abstract

FDG PET/CT often underperforms in characterizing hyper-enhancing, FDG-non-avid renal masses. We present two cases illustrating the potential of ^68^Ga-NY104, a novel small-molecule tracer targeting carbonic anhydrase IX (CAIX), for this differential diagnosis. Both patients presented with a hyper-enhancing right renal mass suspicious for clear cell renal carcinoma (ccRCC) and subsequently underwent both ^18^F-FDG and ^68^Ga-NY104 PET/CT, with histopathology and CAIX immunohistochemistry (IHC) as the reference standard. On ^18^F-FDG, both lesions were non-avid (SUVmax 2.6 and 2.2, Tumor-to-Liver Ratio [TLR] 0.87 and 0.69, respectively). However, on ^68^Ga-NY104 PET/CT, Patient 1 (a 65-year-old man) showed intense, homogeneous uptake (SUVmax 26.0, TLR 4.64), while Patient 2 (a 67-year-old woman) showed negligible uptake (SUVmax 2.5, TLR 0.68). It was consistent with histopathology and IHC results that Patient 1 was CAIX-positive ccRCC, while Patient 2 was CAIX-negative hemangioma. Our preliminary cases suggest the potential utility of CAIX-targeted PET/CT imaging with ^68^Ga-NY104 in differentiating ccRCC from benign mimickers like renal hemangioma, which warrants further prospective evaluation.

**Figure 1:**
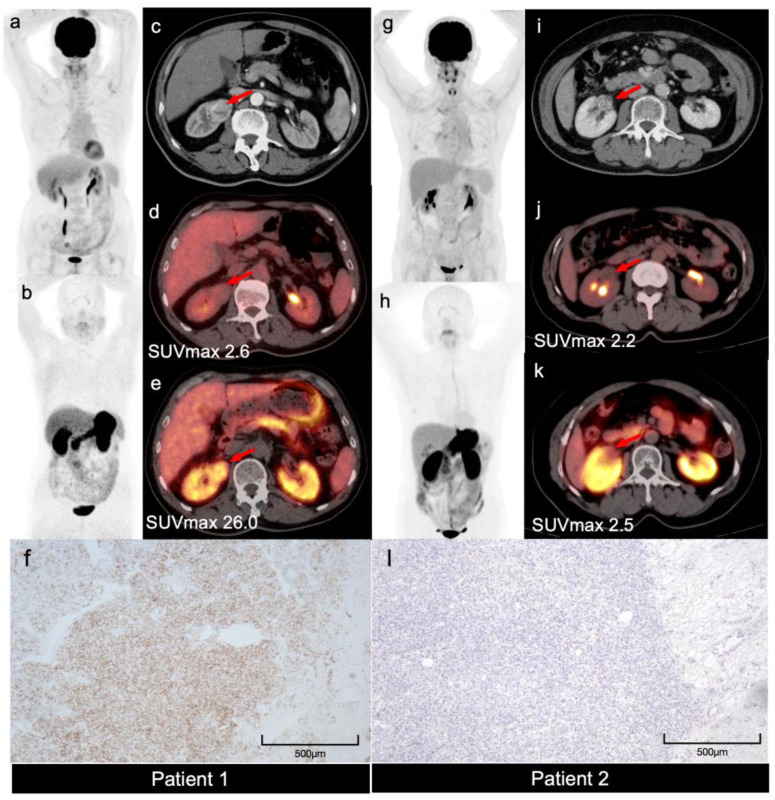
Two patients with right renal mass found during routine examination were presented. For Patient 1 (a 65-year-old man, (**a**–**f**)), contrast-enhanced CT (CECT) revealed a 4.1 cm heterogeneously hyper-enhancing lesion ((**c**), arrow) peaking in the corticomedullary phase (mean attenuation 86.3 HU). For Patient 2 (a 67-year-old woman, (**g**–**l**)), CECT revealed a 2.2 cm lesion that also demonstrated heterogeneous, avid enhancement ((**i**), arrow) peaking in the nephrographic phase (mean attenuation 106 HU). Both lesions raised suspicion of clear cell renal cell carcinoma (ccRCC). For image analysis, scans were performed on a time-of-flight PET/CT scanner (Polestar m680, Sino Union Healthcare Inc., Beijing, China) at 60 min post-injection of approximately 370 MBq ^18^F-FDG or 200 MBq ^68^Ga-NY104. Images were reconstructed using an ordered subsets expectation maximization algorithm (2 iterations, 10 subsets, 192 × 192 matrix) and corrected for CT-based attenuation, dead time, random events, and scatter. Tumor-to-Liver Ratios (TLR) were calculated based on SUVmax. We defined “FDG non-avid” a priori as a TLR < 1.2. PET/CT with ^18^F-FDG (Patient 1, MIP, (**a**), fusion, (**d**), arrow; Patient 2, MIP, (**g**), fusion, (**j**), arrow) was performed. Both lesions were FDG non-avid (SUVmax, Patient 1, 2.6, Patient 2, 2.2; TLR, 0.87 and 0.69, respectively). To further characterize the lesions, PET/CT with ^68^Ga-NY104, a small molecule PET tracer targeting carbonic anhydrase IX (CAIX), was also performed. While Patient 1 showed heterogeneous avid uptake of ^68^Ga-NY104 (MIP, (**b**), fusion, (**e**), arrow, SUVmax 26.0, TLR 4.64), indicating CAIX overexpression, Patient 2 demonstrated minimal, background-level uptake (MIP, (**h**), fusion, (**k**), arrow, SUVmax 2.5, TLR 0.68). Both patients underwent surgery within a month after the PET/CT scans. Patient 1 was confirmed to be ccRCC (WHO Grade 1), while Patient 2 was hemangioma. The CAIX expression was also confirmed using immunohistochemical staining, with diffuse expression in Patient 1 (**f**) and no expression in Patient 2 (**l**). Clear cell RCC (ccRCC) is the most common histological subtype of renal cancer [1,2]. While multiparametric MRI (mpMRI) remains the non-invasive reference, it has known pitfalls in differentiating ccRCC from benign mimickers like oncocytoma and hemangioma [3]. FDG PET/CT, which is often used for staging in other types of cancer [4], has limited sensitivity in detecting RCC due to the relatively low glucose metabolism of RCC cells [5]. CAIX is overexpressed in over 90% of ccRCC due to von Hippel–Lindau (VHL) mutation, making it an ideal target for molecular imaging [6,7,8]. Our cases demonstrated that CAIX-targeted PET/CT imaging using ^68^Ga-NY104 may aid in diagnosing ccRCC and in the differential diagnosis of hyper-enhancing lesions mimicking ccRCC. This tracer contributes to the broader PET landscape for RCC (which includes PSMA [9] and SSTR [10] targets) and may have future theranostic potential in selecting patients for CAIX-directed therapies. However, potential false negatives (in CAIX-negative ccRCC) and false positives (from other hypoxic lesions) should be taken into consideration. Meanwhile, the limitations of this two-case report are clear, including selection bias. Further investigations on other ccRCC mimickers such as oncocytoma, chromophobe, and papillary RCC are warranted. As a novel tracer, ^68^Ga-NY104 is currently for research only and not yet commercially available.

## Data Availability

The raw data supporting the conclusions of this article (including the de-identified DICOM series, anonymized pathology reports, and IHC images) will be made available from the corresponding author on request.
